# A Case of Anaplastic Lymphoma Kinase-positive Large B-cell Lymphoma

**DOI:** 10.4274/tjh.galenos.2019.2019.0064

**Published:** 2019-08-02

**Authors:** Gaurav K. Gupta, Monika Pilichowska

**Affiliations:** 1 Tufts Medical Center, Department of Pathology and Laboratory Medicine, Boston, Massachusetts, USA

**Keywords:** Lymphoma, Non-Hodgkin, Anaplastic lymphoma kinase large B-cell lymphoma

A 48-year-old male presented to the emergency department with a 2-week history of fever, mild pancytopenia, night sweats, and back pain. A computed tomography scan revealed a large retroperitoneal mass (11x6x5 cm). A core biopsy was performed. Hematoxylin and eosin staining from the retroperitoneal mass biopsy showed diffuse atypical lymphoid infiltrate (Figure 1A), composed of large, monomorphic, immunoblast-like atypical cells with vesicular chromatin, prominent central nucleoli, and abundant amphophilic cytoplasm (Figure 1B). Immunohistochemical staining showed that atypical cells were positive for CD45 (weak, arrow; strong positivity in the background lymphocytes, arrowhead) (Figure 1C), CD4 (Figure 1D), CD138 (Figure 1E), MUM1 (Figure 1F), lambda light chain (Figure 1G), EMA (Figure 1H), and anaplastic lymphoma kinase (ALK) immunostain (Figure 1I), showing strong nuclear and cytoplasmic positivity. The atypical cells were negative for CD20, CD79a, CD30, CD3, CD2, CD5, CD7, CD10, and kappa light chain. ALK rearrangement t (2; 5) was identified by fluorescence in situ hybridization, confirming a diagnosis of ALK-positive large B-cell lymphoma (ALK-LBCL). ALK-LBCL is an exceedingly rare and rather recently identified [1] aggressive B-cell neoplasm with approximately 100 cases reported so far in the literature. The diagnosis can be challenging due to the unusual immunophenotype of neoplastic cells and histologic features overlapping with those of other hematologic malignancies.

## Figures and Tables

**Figure 1:**
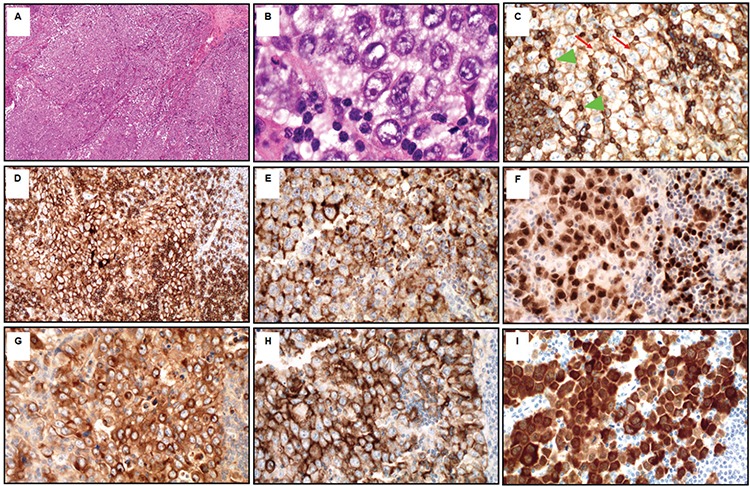
A) Diffuse atypical lymphoid infiltrate by hematoxylin and eosin staining; B) large, monomorphic, immunoblast-like atypical cells with vesicular chromatin, prominent central nucleoli, and abundant amphophilic cytoplasm; positivity of CD45 (C), CD4 (D), CD138 (E), MUM1 (F), lambda light chain (G), EMA (H), and anaplastic lymphoma kinase immunostain (I).
